# Scorpionism: a neglected tropical disease with global public health implications

**DOI:** 10.3389/fpubh.2025.1603857

**Published:** 2025-06-03

**Authors:** Jorge Vasconez-Gonzalez, Harold Alexander-León, María de Lourdes Noboa-Lasso, Juan S. Izquierdo-Condoy, Emilia Puente-Villamarín, Esteban Ortiz-Prado

**Affiliations:** ^1^One Health Research Group, Universidad de las Américas, Quito, Ecuador; ^2^Research Department, SOLCA Núcleo de Quito, Quito, Ecuador; ^3^Faculty of Administration and Public Health, Universidad Peruana Cayetano Heredia, San Martín de Porres, Peru; ^4^Program in Occupational Safety and Health, The University of Porto, Porto, Portugal

**Keywords:** scorpionism, scorpion stings, neglected tropical disease, public health, global health

## 1 Introduction

Scorpions are venomous arthropods belonging to the class *Arachnida* and the order *Scorpiones* (1). To date, ~2,772 species of scorpions have been described globally, of which around 104 species (3.8%) are considered of medical significance due to their venom toxicity and potential for severe envenomation (2). The majority of medically relevant species belong to the family *Buthidae*, which includes genera such as *Androctonus, Buthus, Buthotus, Leiurus, Mesobuthus*, and *Parabuthus*, found in regions such as Asia, India, the Middle East, and North Africa. Additionally, species from the *Tityus* genus are prevalent in South America and the Caribbean, while *Centruroides* species are commonly encountered in Mexico, Central America, and the southwestern United States (3).

Scorpion envenomation, or scorpionism, is recognized as a pressing yet often neglected public health concern, particularly in tropical and subtropical regions. It disproportionately affects rural and socioeconomically disadvantaged communities, where access to timely and adequate medical care is limited (4, 5). The presence of scorpions in human dwellings and workplaces leads to frequent encounters, thereby elevating the risk of envenomation and associated morbidity and mortality (6–8).

Global estimates suggest that over 1.2 million scorpion stings occur annually, resulting in more than 3,000 deaths, with a disproportionately high burden observed in children due to their lower body mass and increased vulnerability to systemic complications (9–11). Mortality is most prevalent in low-resource settings, where delayed access to antivenom and critical care services remains a major barrier. High-incidence countries include Mexico, Brazil, Iran, Algeria, and Morocco, where scorpionism remains endemic and requires targeted preventive and therapeutic strategies (12–15). Table 1 summarizes the number of reported cases, deaths, and incidence rates per 100,000 inhabitants in selected endemic countries and regions.

**Table 1:** Epidemiological characteristics of scorpionism in endemic countries.

**Country**	**Period time**	**Cases**	**Deaths**	**Incidence per 100,000**	**Case fatality rate (%)**	**Main risk groups**	**Most affected regions**	**Predominant scorpion genus/species**	**Access to antivenom**	**Antivenom use recommended?**	**References**
Mexico	Not specified	288,391	32	241.42	0.01%	Children < 15 years	Morelos	Centruroides	Yes	Yes	(2)
Brazil	2022	183.738	92	90.48	0.05%	Adults 20–60 years	Southeast	Tityus	Variable	Yes	(16, 17)
Algeria	2023	46.908	24	99.98	0.05%	15–49 years	Ghardaïa province	Androctonus	Yes	Yes	(18)
Iran	2001–2009	42,500	19	140	0.04%	Adults 20–39 years	Khuzestan and Kohkiloye Boyerahmad	Hemiscorpius	Variable	Yes	(19, 20)
Tunisia	2001	40.000	50	420	0.12%	Children < 15 years	Central	Androctonus	Yes	Yes	(9, 21)
Morocco	2019	26,819	44	753	0.16%	Children < 15 years	Drâa-Tafilalet	Androctonus, Buthus	Yes	Yes	(22, 23)
Saudi Arabia	Not specified	14,00	Not available	90	Not available	15–30 years	Al-Medina Al-Munawar	Leiurus	Yes	Yes	(24)
Türkiye	2005	24,261	0	Not available	0	Not available	Southeaste rn Anatolia	Androctonus, Mesobuthus	Yes	Yes	(25)

This table summarizes scorpion sting cases, mortality, incidence rates, case fatality rates, affected demographics, seasonal patterns, predominant scorpion species, and access to antivenom in countries and regions with significant scorpionism burden.

## 2 Biochemical composition and functional diversity of scorpion venom

Scorpion venom is a mixture of lipids, peptides, enzymes, free amino acids, nucleotides, amines, inorganic salts, mucoproteins, heterocyclic components, and various other substances. The composition of the venom can be divided into toxic and non-toxic fractions (3).

The non-toxic fraction includes enzymes such as hyaluronidases, mucopolysaccharides, histamine, and serotonin (3). These components contribute to the overall toxicity of the venom. For instance, hyaluronidase facilitates the spread of toxins by degrading the extracellular matrix and connective tissues surrounding blood vessels at the site of envenomation (3). Histamine plays a role in mediating the inflammatory response (26).

The toxic fraction is mainly composed of neurotoxins, which are capable of depolarizing the membranes of nerve cells through various mechanisms. For example, β-toxins alter the activation of Na^+^ channels by reducing the peak amplitude of sodium currents and shifting the voltage dependence of channel activation toward more hyperpolarized potentials (27). In contrast, α-toxins inhibit sodium channel deactivation, while κ-neurotoxins block potassium channels (28–30). Meanwhile, calciums act as agonists of ryanodine receptors, increasing intracellular calcium levels (30). Phospholipases disrupt cell membranes by hydrolyzing phospholipids, leading to tissue necrosis and hemorrhage (28, 31). Proteases also play a pivotal role in activating venom toxin precursors through post-translational modifications (3). Proteases are involved in the spread of the toxin through the breakdown of matrix molecules. In addition, proteolytic activity has been observed. Some proteases have been shown to disrupt the transport of pancreatic vesicles to the exterior of the organ, causing pancreatitis (32–34).

Toxins targeting ion channels particularly voltage-gated sodium and potassium channels—are the primary mediators of neurotoxicity and are especially abundant in species such as *Mesobuthus tamulus, Hottentotta saulcyi*, and *Tityus serrulatus* (28, 35, 36). These toxins can account for up to 76.7% of venom mass in some species (35). Moreover, scorpion venom includes a wide variety of pharmacologically relevant peptides and proteins, such as antimicrobial, antiviral, antifungal, and antimalarial peptides (37).

Other notable components of scorpion venom include bradykinin-potentiating peptides and cysteine-rich secretory proteins, which contribute to prey immobilization and immune modulation (31, 38). Non-peptidic molecules like adenosine and citric acid are also present and may act synergistically to enhance the envenomation process (39). Lipid components, including phospholipids and ceramides, are involved in cellular disruption and may modulate inflammation (36).

The inflammatory response triggered by scorpion venom involves a cascade of cellular events and mediator release. Upon envenomation, innate immune cells recognize venom components via pattern recognition receptors, leading to the activation of pro-inflammatory signaling pathways (7, 40). This interaction initiates intracellular signaling cascades that result in the release of inflammatory mediators (40).

The cytokine profile induced by scorpion envenomation varies by species. For example, *Androctonus australis hector* induces the production of IL-1β, IL-4, IL-6, IL-10, and TNF-α; *Tityus serrulatus* induces IL-1β, IL-6, IL-8, IL-10, NO, TNF-α, IL-1α, IL-1β, IFN-γ, and GM-CSF; *Centruroides noxius* induces IL-1β, IL-1α, IFN-γ, IL-6, IL-10, and TNF-α. In the case of *Leiurus quinquestriatus*, the cytokines produced include IL-6, IL-8, NO, and TNF-α (7). These cytokines may be implicated in the pathophysiology of envenomation, such as inflammatory manifestations at the site of inoculation and systemic repercussions, such as pulmonary edema and cardiogenic shock (41, 42).

Despite advances in antivenom therapy, the poor immunogenicity of low molecular mass toxins (3–15 kDa) remains a challenge, as these are often not effectively neutralized by commercial antivenoms (35, 43). Proteomic and transcriptomic studies have revealed a vast diversity in venom composition, which is influenced by species, geographic location, sex, age, and environmental conditions (44). On average, a single scorpion species can produce around 150 different venom components, with ~750 scorpion venom proteins currently cataloged in the UniProt Animal Annotation Program. Toxins are classified based on their ion channel targets: sodium (α-NaScTxs, β-NaScTxs), potassium (α-KTx, β-KTx, γ-KTx, κ-KTx), and calcium (calcines) (27).

Elemental analyses of venom from *Androctonus bicolor, Androctonus crassicauda*, and *Leiurus quinquestriatus* have identified the presence of numerous elements, including sodium, potassium, calcium, copper, germanium, cerium, scandium, lanthanum, silver, gallium, palladium, zirconium, neodymium, bismuth, beryllium, tellurium, thallium, samarium, dysprosium, gadolinium, erbium, cesium, holmium, ytterbium, praseodymium, rhenium, europium, arsenic, manganese, chromium, iron, iodine, selenium, lithium, yttrium, nickel, lead, rubidium, rhodium, uranium, antimony, ruthenium, magnesium, phosphorus, silicon, bromine, aluminum, zinc, strontium, vanadium, barium, and titanium (45). This compositional complexity highlights the need for continued research to improve therapeutic approaches and explore the pharmacological potential of scorpion venom components.

## 3 Risk factors for scorpion stings and severe envenomation

While it is true that one of the main risk factors for scorpion stings is residence in rural areas, where specific environmental and occupational exposures increase the likelihood of encounters. Activities such as storing and handling firewood are particularly hazardous, as they involve prolonged contact with the ground and outdoor environments where scorpions may reside (8). Similarly, the accumulation of leaves, tools, or construction materials near homes provides ideal microhabitats for scorpions, thereby increasing the risk of stings (8).

Notably, some scorpion species exhibit opportunistic behavior, enabling them to adapt to and colonize disturbed or modified environments. These species can establish high-density populations, reproduce rapidly, and survive extended periods without food, allowing them to thrive even under artificial conditions, such as urban settings (14). A prominent example is *Tityus serrulatus*, whose geographic distribution has expanded significantly across Brazil, with notable proliferation in urban areas (46).

Santana and Oliveira reported that the majority of scorpion envenomation cases occurred in urban and peri-urban areas (65.5%) (47). Another large-scale study in Brazil analyzing 20,555 cases of scorpion stings found that most incidents took place in urban settings (*n* = 18,571; 90.35%) (48). Similarly, Furtado et al. (49) observed a high frequency of urban cases (*n* = 9,625; 86.45%) in the state of Ceará, Brazil.

Another relevant risk factor is the presence of birds in the household environment. The straw used in nests serves as a suitable shelter for scorpions, facilitating their proximity to humans (8, 50). Housing conditions also play a critical role. Dwellings constructed with non-durable materials such as tin, wood, or cardboard roofs and dirt floors are more permeable to scorpion intrusion. Additionally, inadequate structural integrity and proximity to natural scorpion habitats further increase the risk of indoor colonization (5, 50). In rural contexts, children frequently assist in agricultural tasks, which further elevate their exposure and risk of stings (50).

Beyond structural and behavioral determinants, several demographic and environmental factors contribute to the epidemiology of scorpionism. Age is a critical determinant of severity, with children particularly those under 15 years being more susceptible to severe envenomation and fatal outcomes (51–53). Occupational exposure remains a significant risk factor, notably among farmers, homemakers, and individuals who handle firewood or rear domestic animals such as ducks and hens (8, 50).

Temporal patterns also modulate risk: stings are more frequent during warmer seasons and nocturnal hours, coinciding with increased scorpion activity (52). Delays in seeking medical care particularly those exceeding 3 h and the inadequate use of antivenom are associated with increased mortality, especially among pediatric populations (51). Emerging evidence has further suggested a possible link between scorpion envenomation and long-term cardiovascular complications, including the development of dilated cardiomyopathy (54).

Inappropriate use of antivenoms remains a critical concern. Fatal outcomes have been associated with improper antivenom administration (51). A study conducted in Brazil involving 293 envenomation cases found that antivenom was inappropriately prescribed in the majority of treated patients (59.7%). Among these cases, the most frequent error was the administration of a higher number of vials than recommended (124 cases; 72.1%), resulting in a total of 323 vials administered beyond protocol guidelines (47). This misuse may stem from the absence of up-to-date reference materials, ineffective dissemination of treatment protocols, and a lack of regular training in the diagnosis and management of scorpion envenomation (47). However, with proper use of antivenom and timely administration, clinical conditions usually improve rapidly, and plasma venom levels generally become undetectable within an hour of starting treatment. Additionally, antivenom reduces the mortality rate even in cases of severe envenomation. It has also been observed that in critically ill children with neurotoxic effects from scorpion envenomation, intravenous administration of scorpion-specific antivenom resolved the clinical syndrome within 4 h (30, 55, 56).

## 4 Public health intervention

### 4.1 Challenges and imperatives in antiserum production

The production and equitable distribution of therapeutic antisera represent a critical, yet often overlooked, component of public health systems particularly in low- and middle-income countries. Despite the availability of technical guidelines and protocols for the manufacture of animal-derived antisera, such as whole IgG or F(ab')_2_ fragments, many public-sector laboratories operate under severe constraints. These include limited production capacity, outdated infrastructure, and insufficient regulatory oversight, all of which hinder their ability to ensure the safety, efficacy, and sustainability of antiserum supply (57, 58).

In Ecuador, the cessation of local snake antivenom production illustrates the vulnerability caused by the absence of long-term planning in serum policy. Historically self-sufficient, the country's sole antivenom manufacturing facility was closed in 2012 amid structural reforms and administrative decentralization, without establishing a sustainable alternative (59). Since then, Ecuador has become entirely dependent on imports for instance, acquiring 42,883 vials from Costa Rica between 2015 and 2017 while facing an increase in snakebite-related hospitalizations and case severity, particularly in remote Amazonian and coastal regions with limited access to emergency care (59).

Another critical issue is the improper storage and transportation of antisera, which can lead to the deterioration or destruction of vials and ampoules. A notable example is the absence of a reliable cold chain, which is essential for preserving the efficacy of liquid antisera. Without appropriate refrigeration, these biological products may become ineffective and unusable (58).

To address global deficiencies in antivenom supply, the World Health Organization (2007) proposed a comprehensive strategy that includes (Table 2):

- Investment in infrastructure and staff training
- Implementation of Good Manufacturing Practices (GMP)
- Strengthening regulatory systems
- Establishment of prequalification programs
- Improved distribution networks guided by epidemiological data
- Continuous clinical training to ensure rational antiserum use

**Table 2:** WHO strategic actions for strengthening antiserum production and distribution systems in public health.

**Strategic area**	**Key actions**	**Expected outcomes**
1. Develop national and regional technical capacity to manufacture antiserums	- Organize workshops focused on Good Manufacturing Practices (GMP), including:	- Enhanced local production capacity
	• Animal handling	- Improved product quality, safety, and efficacy
	• Plasma collection and fractionation	
	• Antigen preparation and storage	
	• Standardized manufacturing protocols	
2. Implement international technological cooperation strategies	- Distribute production responsibilities across laboratories:	- Strengthened global collaboration
	• Some maintain venomous species and prepare high-quality venom pools	- Increased efficiency and specialization
	• Others conduct immunization and plasma processing	- Reduced duplication of efforts
	- Promote knowledge transfer and innovation	
3. Improve logistics and distribution of antiserums	- Design and implement strategic distribution systems	- Improved access in rural and high-risk areas
	- Leverage existing health infrastructure (e.g., vaccine cold chains)	- Reduced product wastage and delays
	- Facilitate exchange of experiences between countries with effective systems	- Enhanced responsiveness to envenomation cases
4. Implement a financially sustainable strategy	- Integrate antiserum production and distribution into national budgets	- Long-term sustainability
	- Promote public-private and international partnerships	- Cost-effective production and delivery
	- Develop affordable and scalable financing models	- Increased trust and investment in public health systems
5. Strengthen regulatory oversight and quality assurance	- Establish or reinforce national regulatory frameworks	- Assurance of product safety and efficacy
	- Implement WHO prequalification schemes	- Elimination of counterfeit or substandard products
	- Standardize potency and safety testing of products	- Increased international confidence in locally produced antiserums
6. Expand training and clinical guidelines for health workers	- Include antivenom administration protocols in medical/nursing curricula	- Reduced misuse of antivenoms
	- Distribute clinical guidelines tailored to local epidemiology	- Improved clinical outcomes
	- Develop e-learning tools and continuous medical education (CME) modules	- Empowered frontline providers, especially in rural settings

One of the most persistent barriers is the lack of coordination between epidemiological surveillance teams and antivenom distribution planners. This misalignment often leads to overstocking in urban hospitals, while rural health posts where most envenomations occur remain under-resourced. Additionally, frontline health workers in these underserved areas frequently lack adequate training in antivenom administration, further exacerbating preventable morbidity and mortality (57, 58).

Ecuador's experience highlights the pressing need to re-establish regional antivenom production capabilities, not only to improve response times but also to enhance health system resilience amid rising envenomation rates. A coordinated regional approach driven by WHO, national governments, and local stakeholders is essential to restore serum availability and reduce the public health burden associated with venomous animal encounters.

Further research is critically needed to evaluate the neutralization efficacy of existing antivenoms against all venomous species endemic to each country. Antivenoms tend to be highly specific to the venom of the species from which they are derived, and cross-neutralization with venom from other species is often limited (60). While in some instances cross-reactivity does occur—as in the case of *A. crassicauda* antivenom (RSHC anti-Ac), which demonstrates immunoreactivity and neutralization potential against *Leiurus quinquestriatus* (61), this is not universally applicable. For example, scorpions of the *Tityus* genus exhibit a high antigenic diversity in their venom, and despite the taxonomic diversity of *Tityus*, only three anti-*Tityus* antivenoms are available in the Americas, targeting *T. serrulatus, T. trivittatus*, and *T. discrepans* (62).

It has been demonstrated that antivenoms often show reduced efficacy outside the native distribution area of the scorpion species for which they were developed. For instance, the Venezuelan anti-*T. discrepans* antivenom does not effectively neutralize the venom of *T. pachyurus* from Colombia, and the Brazilian anti-*T. serrulatus* antivenom is not capable of significantly reversing the severity of cerebellar-muscular symptoms caused by *T. obscurus* (62). The lack of species-specific antivenoms represents a serious public health concern. In Iraq, for example, fatalities occasionally occur due to the unavailability of antivenoms specific to local scorpion species (60).

### 4.2 Role of national authorities in scorpionism prevention and control

#### 4.2.1 Ministry of public health

As the principal agency responsible for protecting population health, the Ministry of Public Health plays a pivotal role in mitigating the public health burden of scorpion envenomation. A fundamental responsibility is ensuring that healthcare professionals particularly those deployed to rural or high-risk areas receive comprehensive training in the clinical recognition and management of scorpion stings prior to assignment (2). This clinical preparedness should be complemented by national public awareness campaigns designed to educate communities on preventive practices, the early signs of envenomation, and the importance of timely medical intervention. These initiatives must be accompanied by robust monitoring and evaluation mechanisms to assess outreach effectiveness and inform policy refinement (2).

At the community level, targeted interventions such as educational workshops can empower local populations to identify medically relevant scorpion species and implement basic environmental control measures. These may include clearing debris, removing weeds, sealing structural gaps, and maintaining household hygiene to minimize scorpion habitats (9, 63). Such community engagement is essential for promoting sustained behavioral change and environmental risk reduction.

Equally critical is the strengthening of epidemiological surveillance systems. Enhanced reporting mechanisms potentially facilitated through mobile health technologies can improve the timeliness and accuracy of sting notifications. Collaborations with local health authorities, civil society, and academic institutions can support the continuous mapping of scorpion species distribution and identify emerging envenomation hotspots (5).

Furthermore, the establishment of national research centers focused on scorpionism would provide the scientific foundation for long-term mitigation strategies. These centers should prioritize interdisciplinary research on venom composition, species ecology, and vector control techniques, thereby facilitating the development of regionally tailored antivenoms and evidence-based interventions (2).

#### 4.2.2 Ministry of public education

Long-term prevention of scorpion stings requires the active involvement of the Ministry of Education through the integration of relevant content into national curricula. Educational programs should include topics on medically important arthropods particularly scorpions to raise awareness among children about envenomation risks, prevention strategies, and the identification of venomous species (2). Instilling these concepts from an early age can reduce the incidence of stings at home and foster a community-wide culture of prevention. It is also important to educate about the importance of ensuring that household waste is properly sealed in plastic bags or other tightly closed containers and is disposed of on the correct day for collection services (64).

Such content should be embedded within science and health education modules and supported by age-appropriate educational materials, including posters, illustrated guides, and digital platforms. To ensure accuracy and engagement, teachers must be adequately trained to deliver evidence-based information tailored to local epidemiological contexts.

Complementary initiatives such as science clubs, school fairs, and interactive workshops can reinforce key prevention messages, especially in endemic regions. By empowering children as health promoters, early education serves not only to protect individual students but also to disseminate knowledge within families and across generations, thereby enhancing community resilience (2).

#### 4.2.3 Ministry of agriculture and environment

The Ministry of Agriculture and Environment holds a pivotal role in reducing scorpion sting incidence through both occupational safety measures and environmental risk mitigation. Agricultural and livestock workers are among the most exposed populations; therefore, the promotion of personal protective equipment such as boots and gloves is essential for minimizing contact with scorpions in high-risk settings (2, 65).

Public awareness campaigns should also address harmful practices such as illegal burning of vegetation, which can disrupt scorpion habitats and inadvertently increase human-scorpion interactions. In parallel, the Ministry should advocate for the safe storage of firewood, crop residues, and construction materials to limit the formation of suitable refuges for scorpions.

To ensure sustained impact, intersectoral collaboration with local governments, academic institutions, and public health authorities is necessary. Joint efforts to map high-risk zones, implement ecological pest control strategies, and integrate environmental sustainability into prevention policies can strengthen the country's capacity to address scorpionism as both a health and environmental challenge (2).

## 5 Conclusion

Scorpionism remains a neglected yet impactful public health issue, disproportionately affecting vulnerable populations in tropical and subtropical regions. Despite its significant global burden, it continues to be overlooked by health authorities and excluded from major international health agendas. Given its epidemiological, social, and economic consequences, scorpionism warrants formal recognition as a neglected tropical disease.

The multifactorial nature of scorpion envenomation shaped by ecological conditions, population vulnerability, health system limitations, and socioeconomic inequalities—demands a coordinated, multisectoral response that integrates environmental management, health education, clinical preparedness, and local antivenom production.

National authorities particularly the ministries of health, education, agriculture, and environment must coordinate efforts to address structural risk factors such as unsafe housing, occupational exposure, and limited access to timely care in rural areas. This includes strengthening regional capacity for antivenom production, improving clinical training in envenomation management, and embedding prevention strategies into school curricula and community programs to foster long-term risk reduction and a culture of awareness and resilience.
